# Torque teno mini virus driven childhood acute promyelocytic leukemia: The third case report and sequence analysis

**DOI:** 10.3389/fonc.2022.1074913

**Published:** 2022-12-15

**Authors:** Xue Chen, Fang Wang, Xiaosu Zhou, Yang Zhang, Panxiang Cao, Xiaoli Ma, Lili Yuan, Jiancheng Fang, Mingyue Liu, Ming Liu, Jiaqi Chen, Qihui Chen, Ping Wu, Yue Lu, Xiujuan Ma, Hongxing Liu

**Affiliations:** ^1^ Division of Pathology & Laboratory Medicine, Hebei Yanda Lu Daopei Hospital, Langfang, China; ^2^ Molecular Medicine Research Center, Beijing Lu Daopei Institute of Hematology, Beijing, China; ^3^ Department of Research and Development, Beijing Geneprofile Technologies Co., Ltd, Beijing, China; ^4^ Department of Bone Marrow Transplantation, Hebei Yanda Lu Daopei Hospital, Langfang, China

**Keywords:** acute promyelocytic leukemia, torque teno mini virus, *RARA*, *TTMV*::*RARA*, whole-transcriptome sequencing

## Abstract

In this manuscript, we report torque teno mini virus (TTMV) as a cause of acute promyelocytic leukemia (APL) lacking *PML*::*RARA* in a 3-year-old boy. Astolfi et al. firstly identified partial integration of the TTMV genome into *RARA* intron 2, which resulted in in-frame *TTMV*::*RARA* fusion in two APL-like pediatric cases without *PML*::*RARA* in November 2021. This fascinating report identified an unexpected exogenous genetic cause of APL and could be of great importance for diagnosing and managing APL. Here we report the third childhood APL-like case caused by TTMV integration and investigate the location and structure of the integrated TTMV sequence. These findings suggest *TTMV*::*RARA* is a recurrent cause of APL lacking *PML*::*RARA*. Considering the widespread prevalence of TTMV in the population, more *TTMV*::*RARA* positive APL-like cases might remain to be identified. Establishing a bioinformatic analysis strategy optimized for the highly variable TTMV genome sequence may facilitate the identification of *TTMV*::*RARA* by whole transcript sequencing. An effective PCR protocol to identify *TTMV*::*RARA* based on a profound analysis of the conservation of TTMV segments in the fusion transcript is also expected. Also, further investigation is needed to elucidate the oncogenic mechanisms of TTMV integration and the clinical features of *TTMV*::*RARA* positive patients.

## Introduction

Astolfi et al. (1, 2) firstly reported torque teno mini virus (TTMV) as a cause of childhood acute promyelocytic leukemia (APL) lacking *PML*::*RARA* fusion. They identified partial integration of the TTMV genome into the intron 2 of the human *RARA* gene, which resulted in in-frame *TTMV*::*RARA* fusion transcripts in two APL-like pediatric cases without *PML*::*RARA* fusion. This is a fascinating report because it identified an unexpected exogenous genetic cause of APL and could be of great importance for diagnosing and managing APL, especially given the widespread recessive carriage and transmission of TTMV in the population.

**Figure 1:**
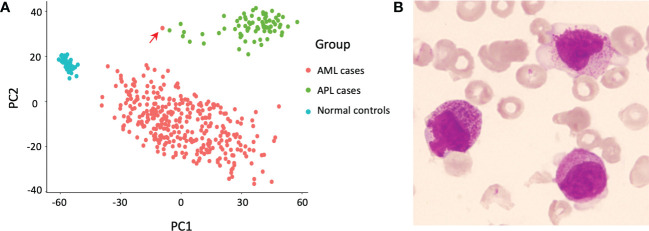
Discovery of the index case. **(A)** Principal component analysis revealed one relapsed acute myeloid leukemia (AML) case (indicated by the red arrow) separate from other AML cases, but adjacent to the cluster of acute promyelocytic leukemia (APL) cases. **(B)** Morphology of the bone marrow (BM) smear at relapse, Wright’s stain.

To explore whether there were more *TTMV*::*RARA* positive APL-like cases, we retrospectively performed a principal component analysis comparing 74 *PML*::*RARA*-positive APL cases, 343 oncogenic fusion gene negative AML cases, and 50 healthy controls in our whole-transcriptome sequencing (WTS) cohort (3). The result showed that one AML case was separated from other AML cases while clustered adjacent to the APL cohort (**Figure 1A**).

## Case description

The index case was a 3-year-old boy initially admitted to a local hospital because of persistent fever and headache. Blood tests showed white blood cell count of 36.76 × 10^9^/L, hemoglobin level of 86 g/L, and platelet count of 117 × 10^9^/L. Prothrombin time and activated partial thromboplastin time were 13.3 s (reference, 9.4 - 12.5 s) and 23.6 s (reference, 25.1 - 38.0 s), respectively. Morphologic examination of bone marrow (BM) smears disclosed infiltration by 73.6% of hyper-granular promyelocytes. Flow cytometry (FCM) revealed 84.1% of myeloblasts (positive for CD13, CD33, CD45, CD117, CD123, CD64, and cMPO; partially positive for CD34; negative for HLA-DR, CD11b, CD16, and other T- or B-lymphoid related markers). The presumptive initial diagnosis of this patient was APL. However, both reverse transcription PCR (RT-PCR) and fluorescence *in situ* hybridization failed to detect the *PML*::*RARA* fusion in the BM, and the karyotype was normal. Next-generation sequencing mutation analysis of 86 genes that are frequently mutated in hematological malignancies revealed *FLT3*-ITD mutation.

Given the clinical suspicion of APL, the patient was immediately treated with ATRA and hydroxyurea. The white blood cell count increased to 128.52×10^9^/L after four-day treatment, and induction chemotherapy (daunorubicin, cytarabine, and etoposide) was started concomitantly. ATRA was discontinued seven days after ATRA initiation due to the absence of *PML*::*RARA*. Follow-up monitoring 21 days after induction chemotherapy showed 13% of myeloblasts and promyelocytes in BM. Then he received consolidation therapy (idarubicin, cytarabine, and etoposide) and achieved complete remission (CR).

However, one month after achieving CR, the patient suffered from recurrent fever and was admitted to our hospital. BM morphology examination showed that 60% of nucleated cells were blast cells with abundant purple granules, irregular nuclei, and cytoplasmatic budding, while negative for Auer rods (**Figure 1B**). The karyotype was normal, and an in-house established protocol of WTS screening for oncogenic fusion genes reported a negative result (3). The patient was started on induction chemotherapy and achieved remission by day 39. Then he underwent haploidentical hematopoietic stem cell transplantation with his father as the donor but relapsed 50 days later with multiple metastases and gave up treatment.

**Figure 2:**
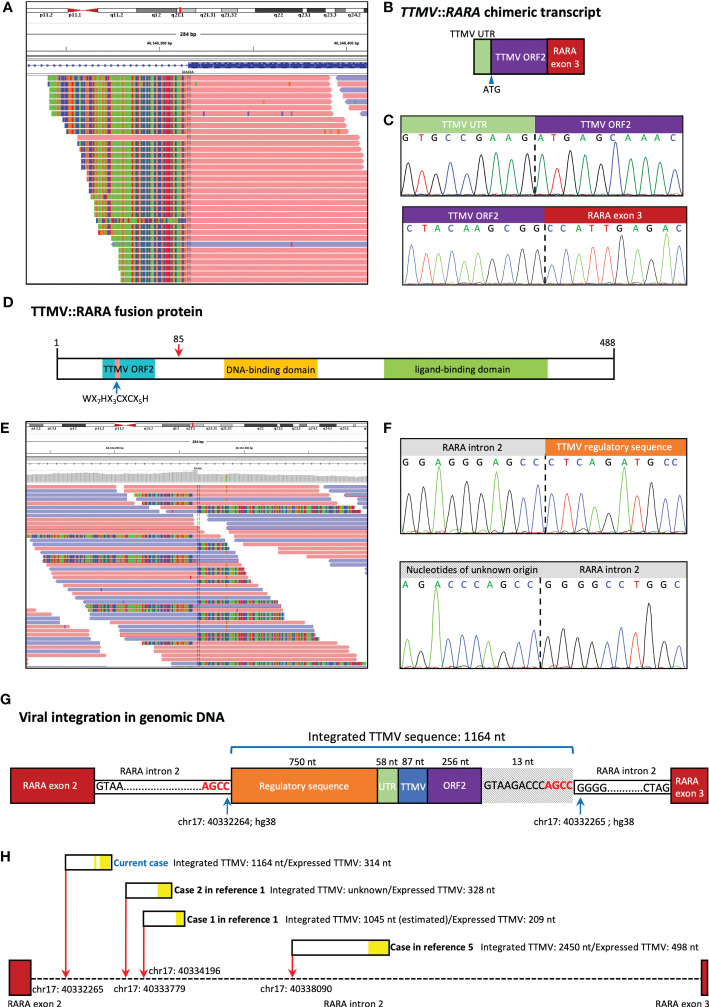
TTMV integration structure of our case. **(A)** Alignment of the whole transcript sequencing (WTS) data in integrative genomics viewer (IGV) revealed abundant *RARA* fusion transcripts with a 5’ extension from *RARA* exon 3, which is not homologous to any human sequence. **(B)** Schematic representation of the fusion between the TTMV sequence and *RARA* exon 3. The chimeric transcript includes TTMV open reading frame 2 (ORF2) and an upstream conserved untranslated region (UTR), in-frame with *RARA* exon 3. **(C)** Sanger sequencing confirmed the existence of the *TTMV*::*RARA* fusion transcript. **(D)** The predicted structure of the TTMV::RARA fusion protein. The blue arrow indicates the highly conserved TTMV ORF2 motif. The red arrow at position 85 indicates the fusion site. **(E)** Whole genome sequencing (WGS) revealed the insertion was located at chr17: 40332265 (hg38) in *RARA* intron 2. **(F)** Sanger sequencing confirmed the two genomic junction sequences. **(G)** Schematic representation of the location and structure of the integrated TTMV sequence. **(H)** TTMV insertion characteristics in the 4 cases reported to date. The red arrows show the insertion positions of TTMV sequence in *RARA* intron 2 (hg38). The black rectangles show the integrated TTMV sequences, and the yellow blocks within them show the nucleotides expressed in the *TTMV*::*RARA* fusion transcript.

Following the retrospective cluster analysis, we performed a manual investigation of the *RARA* transcript sequence in WTS data of this case. Alignment of the WTS data in integrative genomics viewer (IGV) revealed abundant *RARA* fusion transcripts with a 5’ extension from *RARA* exon 3, which is not homologous to any human sequence (**Figure 2A**). Further analysis revealed the exogenous sequence started with 58 nucleotides that aligned to the untranslated region (UTR) of TTMV, followed by 256 nucleotides starting with a start codon (ATG) and revealed a significant alignment to different TTMV isolates (83% coverage, 93% to 94% identity), in-frame with the full *RARA* exon 3. RT-PCR and Sanger sequencing confirmed the existence of the *TTMV*::*RARA* fusion transcript (**Figures 2B, C**). This full-length *TTMV*::*RARA* fusion transcript was predicted to encode a fusion protein containing 488 amino acids (**Figure 2D**). The putative N terminal 85 polypeptide sequence showed 54% identity and 45% coverage to TTMV open reading frame 2 (ORF2) and displayed the conserved motif WX_7_HX_3_CXCX_5_H, which were shared among all anelloviruses. The RARA part of the fusion protein was consistent with *PML::RARA* and all other RARA fusions.

Whole genome sequencing (WGS) and Sanger sequencing confirmed the insertion of 1164 bp exogenous fragment at chr17: 40332265 in *RARA* intron 2, which aligned to parts of the TTMV genome (**Figures 2E, F**). There were 750 nucleotides and 87 nucleotides derived from TTMV genomic sequences upstream and downstream of the TTMV UTR region, respectively. Also, there were 13 nucleotides not belonging to any known TTMV isolates downstream of TTMV ORF2, all of which were removed during splicing (**Figure 2G**). PCR and Sanger sequencing also confirmed the existence of the same genomic fusion sequence in the archived DNA samples at primary diagnosis. The integrated viral sequence possessed the two 15-nt conserved sequences (CGAATGGCTGAGTTT and AGGGGCAATTCGGGC) in the UTR of all TTVs (4). The last four bases (AGCC) of *RARA* intron 2 sequence 5’ to the insertion site were the same as the four 3’ terminal bases of the inserted sequence, suggesting there might be a microhomologous recombination mechanism that mediated the viral sequence integration (**Figure 2G**).

## Discussion

We here report the third childhood APL-like case caused by the integration of TTMV fragment into the *RARA* locus. The first case was a 6-year-old girl diagnosed as hypergranular APL based on the characteristic morphologic features. The patient received CR after treatment combining standard AML induction therapy and ATRA, followed by 3 high-dose cytabine-based courses of consolidation therapy. She relapsed 8 months later and achieved CR after receiving combination of ATRA and ATO. Then she underwent HSCT and was alive at 4 years after HSCT. The second case was a 3-year-old AML child reported in the same literature. *TTMV::RARA* fusion in this patient was identified by a retrospective in silico analysis in a WTS database of 22 pediatric cytogenetically normal AML cases and the laboratory and clinical data were not detailly described (1, 2). Recently, Sala-Torra et al. (5) reported *TTMV*::*RARA* fusion in a 39-year-old man diagnosed as AML with APL characteristics but without *PML::RARA*. He was initially treated with the standard 7+3 regimen and showed induction failure. He then received mitoxantrone, etoposide, and cytarabine with decitabine and venetoclax with a response. **Table 1** provides patient characteristics of all reported cases, including the current case, and the TTMV insertion characteristics in the 4 cases reported to date was summarized in **Figure 2H**. These findings suggest *TTMV*::*RARA* is a recurrent cause of APL lacking *PML*::*RARA* fusion.

**Table 1:** Summary of clinical and laboratory characteristics in the 4 reported cases with *TTMV*::*RARA* fusion.

Literature	Sex	Age (y)	Diagnosis	Initial WBC, ×10^9^/L	Fibrinogen	D-dimer	PT (second)	APTT (second)	Morphology	Immunotyping	Karyotype	RARA break-apart probe	Gene mutation	Treatment	Outcome
2021 Astolfi et al. (1)	F	6	Hypergranular APL	2.8	↓	↑	↑	↑	BM: hypercellularity with 85% abnormal promyelocytes with Aure rods	CD33+, CD13+, CD38+, CD99+, MPO+, HLA-DR^low^	Normal karyotype	RARA rearrangements (-)	NA	ATRA+ATO+chemotherapy+HSCT	CR
2021 Astolfi et al. (1)	NA	3	AML	NA	NA	NA	NA	NA	NA	NA	Normal karyotype	NA	(-)	NA	NA
2022 Sala-Torra et al. (5)	M	39	AML with APL characteristics	NA	↓	↑	↑	NA	PB: circulating promyelocytic blasts with abundant azurophilic granules.	CD33+, MPO+, CD34-, HLA-DR-	46, XY, i(17)(q10)[18]/47, XY, 18, i(17)(q10)[2]	NA	NA	chemotherapy	NA
Our case	M	3	APL	36.76	NA	NA	↑	↓	BM: 73.6% hyper-granular promyelocytes	CD33+, CD13+, CD117, CD64+, MPO+, CD34 partially positive, HLA-DR-, CD11b-, CD16-	Normal karyotype	NA	*FLT3*-ITD	ATRA+chemotherapy+HSCT	Death

AML, acute myeloid leukemia; APL, acute promyelocytic leukemia; APTT, activated partial thromboplastin time; ATO, arsenic trioxide; ATRA, all-trans retinoic acid; BM, bone marrow; CR, complete remission; F, female; HSCT, hematopoietic stem cell transplantation; M, male; NA, not available; PB: peripheral blood; PT, prothrombin time; WBC, white blood cell count; ↑, higher than the reference value; ↓, below the reference value.

Considering the widespread prevalence of TTMV in the population, more *TTMV*::*RARA* positive APL-like cases might remain to be identified. Establishing a bioinformatic analysis strategy optimized for the highly variable TTMV genome sequence may facilitate the identification of *TTMV*::*RARA* by WTS data analysis. An effective PCR protocol to identify *TTMV*::*RARA* is also worth expecting, but this should be based on a profound analysis of the conservation of TTMV segments in the fusion transcript. Also, further investigation is needed to elucidate the oncogenic mechanisms of TTMV integration and the clinical features of *TTMV*::*RARA* positive patients. The first reported case was successfully treated with ATRA and ATO and subsequent HSCT. However, as ATRA was coupled with chemotherapy and HSCT, it is almost impossible to assess the contribution of ATRA. The outcomes of the other two reported cases were not available. In addition, our patient in the current case report died after giving up treatment and the efficiency of ATRA was unclear since ATRA was used only for 7 days. Therefore, further studies into the optimal therapeutic approaches for patients with *TTMV::RARA* are urgently needed.

## Data availability statement

The datasets presented in this article are not readily available because of ethical/privacy restrictions. Requests to access the datasets should be directed to the corresponding author.

## Ethics statement

The studies involving human participants were reviewed and approved by The Institutional Review Board and Ethical Committee of the Hebei Yanda Lu Daopei Hospital. Written informed consent to participate in this study was provided by the participants’ legal guardian/next of kin.

## Author contributions

HL designed the research; XC designed molecular studies and wrote the paper; PC, JF, QC, and XiuM performed bioinformatics analysis; FW, YZ, JC, and MingL supervised clinical and experimental findings; XZ, XiaM, LY, and MingyL performed molecular studies; YL was involved in the management of the patient and provided clinical data. PW performed morphological analysis. All authors reviewed the manuscript and contributed to the final draft.
